# Trade facilitation, market size, and supply chain efficiency of Taiwan semiconductor companies

**DOI:** 10.1371/journal.pone.0299322

**Published:** 2024-10-16

**Authors:** Cheyuan Liu, Tao He, Fangzhou Liu, Shutao Liang, Chunyu Zhang

**Affiliations:** 1 School of Economics and Management, Guangxi Normal University, Guilin, Guangxi, China; 2 Key Laboratory of Digital Empowerment Economic Development (Guangxi Normal University), Education Department of Guangxi Zhuang Autonomous Region, Guilin, Guangxi, China; 3 School of Economics and Finance, Huaqiao University, Quanzhou, Fujian, China; 4 School of Economics and Management, Hezhou University, Hezhou, Guangxi, China; 5 International College, Krirk University, Bangkok, Thailand; Ataturk University, TÜRKIYE

## Abstract

In an environment marked by global economic volatility and geopolitical uncertainties, the stability of Taiwan’s supply chain takes on heightened importance, particularly given Taiwan’s crucial role in the global semiconductor supply chain. In recent years, semiconductor companies in Taiwan have faced increasing inventory pressures, which will reduce their competitiveness and increase operational costs over the long term. Although previous studies have explored the influence of trade facilitation on macroeconomic and trade efficiencies, its specific impacts on the semiconductor industry have been less frequently addressed. This study integrates corporate inventory, trade facilitation, and geopolitical factors within a unified analytical framework to construct a model that explores mediating and moderating effects. This study conducted regression analysis on data from 52 Taiwan-listed integrated circuit companies from 2014 to 2022. Contrary to traditional findings that trade facilitation decreases inventory in other industries, it predominantly fosters inventory accumulation within Taiwan’s semiconductor sector by expanding market size, thereby affecting supply chain efficiency. Moreover, geopolitical factors were found to intensify the effects of trade facilitation on corporate inventory. Elevated geopolitical risks lead to greater inventory accumulation, which ultimately threatens long-term competitiveness and diminishes the semiconductor industry’s advantage in Taiwan, further influencing supply chain efficiency. Consequently, this study recommends that to more accurately forecast market size, semiconductor companies in Taiwan are encouraged to expand their manufacturing investments in Chinese mainland. Additionally, the prudent handling of cross-strait relations by the Taiwan authorities is an important strategy to mitigate geopolitical risks affecting the semiconductor supply chain.

## 1. Introduction

In the contemporary global economic and technological landscape, the semiconductor industry is pivotal. The industry is characterized by rapid technological advancements and high product specialization, resulting in a complex, extensive global supply chain network. According to the 2022 financial reports of major semiconductor companies like Intel, AMD, Samsung, and TSMC, significant inventories are maintained.(https://epsnews.com). Intel’s annual report underscores that excessive inventory leads to increased holding costs and a significant reduction in gross margins, which impairs supply chain efficiency. Taiwan plays a critical role in the global semiconductor supply chain, holding 73% of Asia’s semiconductor foundry market share(https://crsreports.congress.gov), which represents 80% of the global market as of 2020(https://www.whitehouse.gov).

Research on semiconductor companies’ supply chain efficiency and inventory management has primarily focused on three perspectives. Firstly, the information-sharing perspective highlights the impact of supply-demand uncertainty on inventory, suggesting that enhanced market size awareness can mitigate excess inventory and boost supply chain efficiency [1–3]. Secondly, the infrastructure construction perspective emphasizes that robust software and hardware infrastructure can reduce transportation and inventory costs, thereby enhancing overall supply chain efficiency [4–6]. Thirdly, the transportation planning, supply chain networks, and management perspective aims to improve transportation efficiency, optimize supply chain structures, and enhance supply chain management [7–13].

Semiconductor companies, with their lengthy manufacturing cycles, short product lifecycles, and complex supplier networks, face increased safety and finished goods inventories, making them susceptible to geopolitical factors [14–17]. Thus, the formation and impact of their inventories are marked by fragility [15], structural inefficiency [18], and significant bullwhip effects [19].

As international economic conditions shift, Taiwan semiconductor exporting companies increasingly face heightened uncertainty. Challenges posed by inventory costs hinder their deep engagement in high-quality international trade. Trade facilitation, which aims to reduce trade costs and enhance efficiency, transparency, and predictability(https://www.wto.org/), is essential for improving supply chain efficiency. Therefore, enhancing trade facilitation, reducing trade costs, and standardizing trade procedures are crucial for improving supply chain efficiency.

Scholars selecting trade facilitation indicators often reference Wilson’s system [20, 21], which quantitatively evaluates aspects like port infrastructure, customs environment, institutional environment, and e-commerce levels. In constructing trade facilitation systems, scholars have employed methodologies like principal component analysis [22, 23], the entropy method [24], and Analytic Hierarchy Process (AHP) [25, 26] to weight selected indicators. Others have utilized simple averaging methods for comprehensive indicator calculation [21, 27, 28].

Current research predominantly focus on national or regional data often overlooking detailed analyses of trade facilitation in Taiwan’s counties and cities. This study employs Wilson’s indicator system [20, 21] to assess trade facilitation levels across Taiwan and explores their impact on the supply chain efficiency of the semiconductor industry.

The New-New Trade Theory suggests that a company’s export decisions hinge on both fixed and variable costs [29]. Reducing these costs can directly and indirectly lower inventory costs and enhance supply chain efficiency. Implementing trade facilitation reforms is vital for this purpose [6, 30]. However, the current research on trade facilitation’s impact on semiconductor inventories is sparse and requires further exploration.

For semiconductor companies in Taiwan, it is crucial to determine whether further enhancements in trade facilitation levels will affect their inventory management and whether geopolitical factors influence this impact? Identifying the causes behind these differentiated impacts is crucial. This study explores strategies to enhance semiconductor companies’ supply chain efficiency in this context.

This study contributes in two ways: Firstly, unlike previous literature that supports the reduction of corporate inventory through trade facilitation, this study empirically demonstrates that enhanced trade facilitation levels promote inventory growth in semiconductor companies in Taiwan and confirms the mediating role of market size in this process. Secondly, using a moderating effect model, the study quantitatively analyzes the increasingly significant impact of geopolitical factors on the semiconductor industry, validating that the tense geopolitical situation in Taiwan following the U.S.-China trade war intensifies the inventory-promoting effect of trade facilitation on semiconductor companies. The analysis based on market size and geopolitical factors provides new insights for semiconductor companies in Taiwan to improve supply chain efficiency.

This study is structured as follows: It first clarifies the relationship between trade facilitation, market size, geopolitical risk for Taiwan, and corporate supply chain efficiency. It then details the model design, variables, and data, followed by an analysis of the proposed research model. The study concludes by highlighting its theoretical and practical contributions to enhancing the supply chain efficiency of semiconductor companies in Taiwan and addresses research limitations and future suggestions.

## 2. Literature review

### 2.1 Trade facilitation and semiconductor company inventory levels

Trade facilitation, comprising a series of institutional norms designed to reduce trade costs, strives to harmonize specific standards internationally to enhance efficiency, transparency, and predictability based on established norms, standards, and globally recognized timeframes [21]. The level of trade facilitation has been quantitatively analyzed from four perspectives: port infrastructure, customs environment, institutional environment, and e-commerce level [31].

In modern manufacturing, supply chain efficiency is a paramount performance dimension [32], characterized as the process of delivering products of the right quality to the correct location at the optimal time with minimal costs [33]. It encompasses not only the overall costs of the supply chain but also aspects such as delivery cycle performance [34], delivery timeliness [35], and inventory levels [36]. Past research has assessed supply chain efficiency by examining changes in corporate inventory indicators, employing approaches like "Just-In-Time" and lean management to significantly elevate profits through quality enhancement, inventory reduction, minimizing physical redundancy, and achieving considerable cost savings [37]. Thus, for export companies, effective inventory management and cost reduction are fundamental to boosting supply chain efficiency.

Trade facilitation, a crucial topic in international trade, is intimately connected with corporate supply chain efficiency. Enhanced trade facilitation serves as an external catalyst for export companies to improve their supply chain efficiency. The New-New Trade Theory posits that a company’s export decisions are swayed by both fixed costs of entering international markets and a company’s variable costs [29]. Undoubtedly, improving trade facilitation measures can lower fixed costs associated with communication and transportation, as well as variable costs related to procurement, thus either directly or indirectly diminishing inventory costs for export companies, promoting lean management, and bolstering supply chain efficiency. Existing research confirms that trade facilitation across the industrial sector leads to reduced corporate inventory levels [28], highlights the positive influence of information technology capabilities on corporate inventory efficiency [38], and shows that trade facilitation transportation infrastructure can curtail corporate transportation expenses [6]. Nevertheless, empirical evidence from the semiconductor industry remains scant, prompting this study to propose the following hypothesis:

H1: Trade facilitation will have a negative impact on the inventory levels of semiconductor companies.

### 2.2 The mediating role of market expansion

Inventory management is intimately linked with economic scale and operational efficiency [39, 40]. Concurrently, a reduction in inventory, driven by the need to minimize inventory costs, serves as a crucial indicator of enhanced supply chain efficiency.

Robust trade facilitation infrastructure and systems are pivotal for ensuring the seamless movement of export products in international trade. Studies indicate that elevated trade facilitation levels can lead to reduced trade costs and broaden market demand for products [41, 42]. In the context of deeply integrated global value chains, the significance of trade facilitation is further magnified amidst geopolitical uncertainties and similar challenges.

The classical inventory control model, known as the EOQ model, posits that a company’s inventory determination hinges on two principal factors: the scale of product sales and the lead time, which is defined as the interval between placing an order and delivering the goods [43, 44]. Trade facilitation impacts corporate inventory decisions by influencing product market demand. Nevertheless, amidst profound shifts in the global economic landscape, geopolitical uncertainties can disrupt supply chains and regional trade [45], thereby impacting inventory control in companies and resulting in diminished supply chain efficiency.

In examining the influence of market size, the impact of the internet and informatization on the semiconductor industry is critical. Digitalization considerably reduces the cost of information searching and processing and adds more dynamics to industrial chain embeddedness [46]. Within the rapidly evolving digital environment, the lifecycle of semiconductor products is noticeably shortening, and product diversity is concurrently expanding, giving rise to increasingly segmented and varied market demands [16, 18, 47]. This diversification inevitably leads to heightened corporate inventory levels [48]. Research conducted by PEI [49] demonstrates that the internet not only fosters numerous niche demand markets but also encourages the "long tail effect" in businesses, characterized by enhanced flexibility, differentiation, a wide variety of products, and small-scale production. This effect presents new competitive advantages for small and medium-sized enterprises (SMEs), particularly in producing mid-to-low-end chips.

The influence of trade facilitation on corporate exports is a significant factor that cannot be overlooked. Research indicates that trade facilitation markedly impacts the expansion margin of corporate exports, particularly for companies exporting differentiated products, which exhibit a more pronounced response to trade facilitation measures aimed at reducing trade barriers [50]. It is thus deducible that enhancements in trade facilitation levels are likely to bolster the export expansion margin of semiconductor products. Such a development is poised to aid medium and small-scale semiconductor companies in better adapting to and meeting market demands, especially those engaged in mid-to-low-end chip manufacturing, and is anticipated to contribute to broader market scale expansion. Additional studies reveal that the market size pathway has a more substantial effect on corporate inventory in firms producing differentiated products [51], and that an increase in product variety is positively associated with corporate inventory [52]. Consequently, with the ongoing evolution of the internet and information technology, coupled with the progress in global trade facilitation, medium and small-scale semiconductor companies are set to encounter unparalleled market opportunities and a corresponding rise in inventory levels.

This suggests that improvements in trade facilitation levels are poised to increase the export expansion margin of semiconductor products, thereby enabling medium and small-scale semiconductor companies to align more closely with market demands. This enhancement particularly benefits mid-to-low-end chip manufacturers in expanding their market reach and potentially raises the inventory levels of semiconductor firms. In the semiconductor industry, market expansion emerges as a more dominant pathway influencing corporate inventory compared to the transportation cost pathway of trade facilitation. Notably, the industry’s supply chain is characterized by limited elasticity; wafer fabrication plants entail substantial investment, and the semiconductor production cycle is lengthy, averaging 26 weeks from wafer input to the production of integrated circuit finished products(https://www.semiconductors.org). When rapidly growing market demands outpace existing supply capabilities, it results in inefficient production and delays in order fulfillment, leading to the accumulation of non-finished product inventory and diminished corporate supply chain efficiency. Consequently, this study posits the following hypothesis:

H2: Trade facilitation indirectly impacts corporate inventory levels through market expansion.

### 2.3 The moderating role of geopolitical factors

Amid the current profound shifts in the global economic landscape, geopolitical tensions are heightened, marked by ongoing trade frictions. Post-1980s, escalating trade frictions between Japan and the US inflicted significant damage on Japan’s semiconductor integrated circuit industry [53]. Similarly, frequent China-US trade disputes have precipitated a substantial contraction in semiconductor product exports across the East Asian region, critically affecting the semiconductor supply chain’s stability and corporate investment strategies [54–56]. Prior research indicates that TSMC has been adversely impacted in the escalation of China-US rivalry [57], with geopolitical factors prompting supply chain restructuring and reconfiguration within mobile phone production networks [45].

This section examines the moderating influence of geopolitics. It argues that the effect of trade facilitation measures on supply chain efficiency (specifically corporate inventory) varies according to the geopolitical context of the region. Geopolitical risks, which significantly influence socio-economic and long-term business environments, can either enhance or reduce these economic effects on companies. Drawing on Yaoqi Guo’s (2023) research [58], the paper categorizes geopolitical relations into two scenarios: geopolitical easing and geopolitical tension. It then explores how geopolitics moderates the impact on semiconductor companies’ inventory from both perspectives.

#### 2.3.1 Impact of geopolitical easing on the semiconductor supply chain

Geopolitical easing is characterized by cooperation initiatives, such as signing trade agreements and international capital investments [59]. Trade agreements, like RCEP (Regional Comprehensive Economic Partnership), contribute to the stability of upstream supplies, including chips and textiles [60]. Such agreements can engender technological spillover and facilitate access to high-tech products for lower-tech regions [59], thus reducing uncertainties in the semiconductor supply chain. Additionally, geopolitical stability attracts significant international capital, which expands market size. A Stable geopolitical environment positively influence environmental finance by encouraging foreign investments and technology trade, speeding up economic development [61]. Conversely, geopolitical tensions result in capital flight [62, 63]. Consequently, geopolitical easing can enhance the effects of trade facilitation measures, leading to increased investments and possibly larger inventories due to market expansion.

#### 2.3.2 Impact of geopolitical tension on the semiconductor supply chain

Geopolitical tension, as manifested in conflicts, wars, sanctions, and technological blockades, will disrupt trade throughout all stages of the industrial chain [58]. This may lead to higher transportation costs and the need for increased safety inventories. Geopolitical unrest can disrupt supply chains, damage infrastructure, and inflate operational costs, adversely affecting financial performance and sustainability [64]. Geopolitical tensions have elevated expenses in reshoring supply chain processes, particularly in regions like Taiwan, South Korea, and Japan [65]. The above factors may weaken the impact of trade facilitation on reducing inventory costs for enterprises, thereby forcing them to prepare more safety stock.

Consequently, this study posits the following hypothesis:

H3: Geopolitical factors will act as a moderator in the influence of trade facilitation factors on corporate inventory.

## 3. Research method

### 3.1 Model design

#### 3.1.1 Mediating effect model

Drawing on the inventory determination model and referencing seminal research [51, 66–68], this study formulates the following baseline model to assess the impact of trade facilitation on the inventory of Taiwan integrated circuit companies:

$${Inventory}_{idt}=\beta_{0}+\beta_{1}{TFI}_{dt}+\beta X_{idt}+\sigma_{i}+\sigma_{t}+\varepsilon_{idt}$$ (1)

In this equation, *i* denotes the company, *d* indicates the division of the county or city where the company’s integrated circuit is situated, *t* represents the year, *Inventory*_*idt*_ is the non-finished product inventory of the company’s industry, *TFI*_*dt*_ is the level of trade facilitation in the region where the company belongs, *X*_*idt*_ encompasses the collection of control variables at the company and regional levels, *σ*_*i*_ is the company fixed effect, *σ*_*t*_ is the time fixed effect, and *ε*_*idt*_ is the random disturbance term. The value of *β*_1_ reflects the degree to which trade facilitation influences the company’s inventory.

Furthermore, to examine if trade facilitation impacts company inventory via the market expansion pathway and to test Hypothesis 2, the study employs a mediating effect regression approach. The following regression is conducted based on the baseline model:

$${markets}_{dt}=\beta_{0}+\beta_{1}{TFI}_{dt}+\beta X_{idt}+\sigma_{i}+\sigma_{t}+\varepsilon_{idt}$$ (2)

$${Inventory}_{idt}=\beta_{0}+\beta_{1}{TFI}_{dt}+\beta_{2}{markets}_{dt}+{\beta X}_{idt}+\sigma_{i}+\sigma_{t}+\varepsilon_{idt}$$ (3)

In Eq (2), *markets*_*dt*_ is posited as a mediating variable. This study scrutinizes the variation in the coefficient of *β*_1_*TFI*_*dt*_ across models (1) and (3). The presence of a partial mediating effect is indicated if the coefficient of *TFI*_*dt*_ in model (3) remains significant but diminishes. Conversely, a non-significant coefficient of *TFI*_*dt*_ combined with a significant coefficient of the mediating variable *markets*_*dt*_ denotes a complete mediating effect.

#### 3.1.2 Moderating effect model

This study examines whether the influence of trade facilitation on supply chain efficiency is altered under geopolitical pressures and aims to validate Research Hypothesis 3. Drawing on Chu [61], it introduces the moderating variable *political*_*t*_ into the baseline model, representing the geopolitical factors contributing to shifts in the current global economic climate. The study constructs an interaction term, *gpr*_*twn*_*t*_×*TFI*_*dt*_, to assess the impact of geopolitical factors on the efficacy of trade facilitation. A significant coefficient for this interaction term would signify the presence of a moderating effect.


$${Inventory}_{idt}=\beta_{0}+\beta_{1}{TFI}_{dt}+\beta_{2}{gpr\_twn}_{t}+\beta_{3}{gpr\_twn}_{t}\times {TFI}_{dt}+X_{idt}+\sigma_{i}+\sigma_{t}+\varepsilon_{idt}$$ (4)

### 3.2 Variable description

#### 3.2.1 Dependent variable

In this study, the dependent variable is company inventory, as referenced in other studies [66, 67, 69]. The non-finished product inventory of the company has been selected to represent company inventory. This choice is grounded in two main considerations: firstly, the classical inventory determination theories cited in this study predominantly focus on non-finished product inventory; secondly, the data collected reveal that non-finished product inventory comprises approximately 72% of the total inventory in integrated circuit companies, serving as the primary source of inventory fluctuations. Therefore, the term ’inventory’ used henceforth in this paper pertains to non-finished product inventory, quantified in thousands of New Taiwan Dollars and log-transformed for inclusion in the equation. The companies under study are publicly listed firms within the semiconductor industry, as identified in the Taiwan Public Information Observation Station, with stock codes and names detailed in the annex. The financial data spans the years 2014–2022.

#### 3.2.2 Explanatory variables

The primary explanatory variable in this study is trade facilitation. Informed by the work of Wilson [20, 21], the study constructs trade facilitation indicators encompassing four domains: customs environment, port efficiency, transportation infrastructure, and e-commerce. Initially, a scoring method and proxy variables are employed to evaluate the performance in these areas of trade facilitation. Given the differences in statistical methods across various sub-indicators of trade facilitation, this study standardizes these sub-indicators using the method outlined in Fan [70]. The sub-indicator values range from 0 to 10, with higher scores indicating more advanced levels of trade facilitation. Subsequently, the standardized data are aggregated and averaged to derive the trade facilitation index for each region where Taiwan integrated circuit companies operate. For detailed information on the construction and selection of specific indicators, refer to Table 1.

**Table 1:** Construction table of trade facilitation indicators by science park and county/city.

Variable	Approach	Variable type	Variable source
Port efficiency X1	Ratio of port trade volume to total trade volume (data of top three industrial parks)	Positive	Taiwan Technical Committee for Scientific Affairs
Transport infrastructure X2	Road mileage intensity (km/km2) by counties and cities	Positive	Taiwan Statistics Bureau for Economic Affairs
Customs environment X3	Offender rate: (person/100,000 persons) by counties and cities X4	Negative	Taiwan Statistics Bureau for Economic Affairs
	Number of detected economic cases:(cases) by counties and cities X5	Negative	Taiwan Statistics Bureau for Economic Affairs
E-commerce X6	Internet usage rate by counties and cities X7	Positive	National Communications Commission
	Balance held on deposit in financial institutions by counties and cities X8	Positive	Banking Bureau, Financial Supervisory Commission
	Balance held on loan in financial institutions by counties and cities X9	Positive	Banking Bureau, Financial Supervisory Commission

Note: X3 is derived from the average summation of x4 and x5; X6 is obtained by averaging and summing x7, x8, and x9. The measurement methods for port efficiency and transportation infrastructure refer to Duan [28], while the customs environment and e-commerce measurement methods refer to Shepherd [71], Mann [27] and Luo [72]. Conceptually similar indicators are selected for calculation. Considering that Wilson [21] and Duan [28] used a general average method to weight and aggregate sub-indicators, this construction also adopts the general average method. The trade facilitation index (tfi) is derived by averaging x1, x2, x3, and x6. In the robustness test, the entropy method is used for aggregation.

The mediating variable in this study is market size. Given that all Taiwan semiconductor companies are located within three science parks in Taiwan, the annual total trade volume of these parks is chosen as the proxy variable for market size. As for the moderating variable, it is selected based on the geopolitical risk index constructed by Caldara, Dario and Matteo Iacoviello [63]. This rating serves as an indicator of the impact of geopolitical relations on the semiconductor industry.

#### 3.2.3 Control variables

In assessing company inventory, individual company differences significantly influence inventory levels. This study, drawing upon DUAN & JING [28], includes key control variables such as total factor productivity (tfp), company sales scale (scale), research and development expenditure (R&D), profit margin (profit), and capital intensity (ci) [28]. Total factor productivity is determined using the DEA-Malmquist index method [73], utilizing data from the Taiwan Stock Exchange. The selection of variables involves three inputs—non-current assets, number of employees, operating costs—and two outputs: annual revenue and net profit. Company sales scale (scale) is gauged using corporate revenue data from the Market Observation Post System. Research and development (R&D) expenditure is ascertained from each company’s financial statements [74]. Profit margin (profit) is calculated as net profit divided by current operating income. Capital intensity (ci) is assessed by the logarithm of the ratio of a company’s fixed assets to its number of employees. In the absence of a fixed assets account in Taiwan’s accounting statistics, the ratio of real estate, plant and equipment to the number of employees is used as a substitute.

As shown in Tables 2 and 3, explanation and descriptive statistics for key variables.

**Table 2:** Explanation of key variables.

Variables	Variable symbol	Variable type	Variable description
Business inventory of non-finished products	inventory	Core explained variable	MOPS
Trade facilitation level	tfi	Core explanatory variable	Constructed by this study
Total factor productivity	tfp	Control variable at the individual level	Measured and calculated by this study
Sales scale	scales	Control variable at the individual level	MOPS
Profit rate	profit	Control variable at the individual level	MOPS
Capital intensity	ci	Control variable at the individual level	MOPS
Research and development expenditure	rd	Control variable at the individual level	MOPS
Regional market size	markets	Mediating variable	Taiwan Technical Committee for Scientific Affairs
Gross output of the electronic component manufacturing industry	ic_a	Mediating variable	Taiwan Statistics Bureau for Economic Affairs
Average number of days sales in inventory	lnvday	Explained variable	MOPS
Geopolitical risk index for Taiwan	Gpr_twn	Moderating variable	Constructed by Caldara, Dario and Matteo Iacoviello (2022)

Note: For the calculation of Total Factor Productivity (tfp) of companies, the data is sourced from the Taiwan Stock Exchange. This study follows the approach of X. Chen [75] and Wu [76], using the DEA-Malmquist index method for calculation.

**Table 3:** Descriptive statistics of key variables.

Variable	Obs	Mean	Std. dev.	Min	Max
ln(inventory)	468	13.309	1.748	7.641	18.865
ln(tfi)	468	1.696	0.297	0.676	2.056
ln(tfi_1)	468	-1.332	0.501	-2.773	-0.110
tfp	468	1.025	0.131	0.381	2.088
ln(scales)	468	15.025	1.981	10.115	21.792
profit	468	0.130	0.431	-5.351	2.613
ci	468	7.646	1.069	3.045	10.683
ln(rd)	468	13.067	2.094	4.466	18.911
ln(markets)	468	9.487	0.450	8.020	10.152
ln(ic_a)	468	22.201	0.164	22.064	22.539
ln(invday)	467	4.429	0.615	2.192	6.411
gpr_twn	468	0.092	0.094	0.014	0.317

Note: Some missing values in financial data are supplemented in forms of linear interpolation. The symbol of “ln()” between the variable means the logarithm of this variable.

## 4. Empirical analysis

### 4.1 Basic regression

This study initiates by exploring the relationship between trade facilitation and inventory, employing regression analysis while controlling for double fixed effects—both individual and temporal—referencing Duan [28]. Table 4 displays the baseline model’s regression outcomes. To elucidate the dynamics between trade facilitation and corporate inventory, the primary explanatory variables are initially regressed. Column (1) demonstrates that the trade facilitation coefficient is positive and achieves statistical significance at the 5% level. Subsequent to the inclusion of control variables and double fixed effects in Column (2), trade facilitation coefficient remains positive and significant at the 10% level. In Column (3), considering the presence of heteroskedasticity and cross-sectional correlation in the data, the regression results using Driscoll Kraay standard errors are reported and analyzed as a standard method.

**Table 4:** Basic regression.

VARIABLES	(1)	(2)	(3)
	Ln(inv)	Ln(inv)	Ln(inv)
Ln(tfi)	0.657**	0.688*	0.688***
	(0.271)	(0.380)	(0.181)
tfp		-0.252	-0.252
		(0.180)	(0.137)
Ln(scales)		0.271***	0.271***
		(0.0602)	(0.0613)
Profit		0.180***	0.180*
		(0.0615)	(0.0824)
Ci		-0.296***	-0.296**
		(0.0595)	(0.0899)
Ln(rd)		0.625***	0.625***
		(0.0571)	(0.0910)
Constant	12.19***	2.429**	2.429
	(0.467)	(1.048)	(1.531)
Time Fixed Effects	No	Yes	Yes
Individual Fixed Effects	No	Yes	Yes
Observations	468	468	468
R-squared	0.012	0.504	
Number of groups		52	52

Note: Standard errors in parentheses;

*** p<0.01,

** p<0.05,

* p<0.1.;

The symbol of “ln()” between the variable means the logarithm of this variable.

Based on Column (3) results, each 1% increase in trade facilitation corresponds to an average inventory increase of 0.688%, a relationship statistically significant at the 1% level. This indicates that trade facilitation enhancements effectively raise average corporate inventory levels. Regarding other control variables, lower capital intensity suggests that a company is likely a small or medium-sized labor-intensive manufacturer, such as those in semiconductor packaging and testing, which may result in higher inventory levels. Conversely, higher research and development expenditures indicate that a company is developing a wider range of new products, necessitating increased inventory to accommodate the demands of new product development and production processes. This aligns with the reasoning behind Hypothesis 2. Although total factor productivity of companies shows a negative correlation with inventory levels, it failed to reach statistical significance. Corporate profit margins positively correlate with inventory levels, contradicting Hypothesis 1. This discrepancy suggests that the effects of trade facilitation on corporate inventory are complex and not merely linear. For instance, Duan [28] notes that trade facilitation can reduce corporate procurement costs by shortening lead times and reducing uncertainties, potentially lowering inventory if market demand remains constant. Conversely, in a manner analogous to the delivery cost effect described by Wan [69] for road infrastructure, considering market demand fluctuations, trade facilitation might similarly reduce export costs and increase the scale of exports, thereby increasing inventory demand. In scenarios where output delivery cost effects predominate, companies may maintain higher inventory levels to manage significant demand shocks, maximize profits, and sustain production.

Therefore, the estimates in Column (3) support Wan [69]’s finding, indicating that for the semiconductor industry in Taiwan, reductions in output delivery costs due to enhanced trade facilitation level likely expose Taiwanese semiconductor companies to larger markets. The study will next examine the effects of variables such as market size, company type, and geopolitical risks to confirm the robustness of these conclusions.

### 4.2 Robustness test

#### 4.2.1 Replacement of key variables

This study assesses the robustness of the baseline regression by modifying key variables. Initially, it substitutes the dependent variable, using average days of inventory (invday) instead of company’s non-finished product inventory. A prolonged inventory duration inversely correlates with the company’s supply chain efficiency. The regression results displayed in the Column(1) of Table 5 demonstrate that for every 1% increase in trade facilitation level, the average inventory days of enterprises increase by 0.769%, and this relationship is statistically significant at the 1% level, affirming the robustness of the initial regression findings.

**Table 5:** Robustness test.

VARIABLES	(1)	(2)
	Driscoll_Kraay_3	Driscoll_Kraay_5
	Ln(invday)	Ln(invday)
Ln(tfi)	0.769***	
	(0.160)	
Ln(tfi_1)		2.379*
		(1.036)
tfp	-0.126	-0.139
	(0.196)	(0.196)
Ln(scales)	0.0585**	0.0556**
	(0.0193)	(0.0218)
profit	-0.0735	-0.0660
	(0.0401)	(0.0396)
ci	-0.134**	-0.121**
	(0.0458)	(0.0460)
Ln(rd)	0.126*	0.126*
	(0.0576)	(0.0554)
Constant	1.685**	2.250***
	(0.512)	(0.521)
Time Fixed Effects	Yes	Yes
Individual Fixed Effects	Yes	Yes
Observations	467	467
Number of groups	52	52

Note: Standard errors in parentheses;

*** p<0.01,

** p<0.05,

* p<0.1;

The symbol of “ln()” between the variable means the logarithm of this variable.

This study subsequently adjusts the explanatory variable, taking into account entropy differences within the dataset. The level of trade facilitation is re-evaluated using the entropy method, following the approach suggested by Shan [24]. The indicators in the baseline regression model are then substituted with new explanatory and dependent variables for further regression analysis. As demonstrated in Column (2) of Table 5, the results indicate that each 1% increase in trade facilitation corresponds to the average inventory days of enterprises increase by 2.379%, a relationship statistically significant at the 1% level. These results uphold the robustness of the baseline regression, exhibiting consistent outcomes even after substituting variables. This consistent performance further substantiates the reliability of the baseline model’s conclusions.

#### 4.2.2 Subsample regression

K.-J. Wang [77] notes that companies in the design phase of semiconductor product production typically produce only a limited number of ’defective products’, resulting in lower inventory pressure. In contrast, companies involved in the production phase face increased pressure due to substantial accumulation of non-finished product inventory during the IC manufacturing process, as also noted by Hwang [77, 78]. Therefore, inventory pressures vary significantly across different stages of the semiconductor supply chain. This study classifies companies based on their production stages and performs regression analysis. Integrated circuit companies are categorized into design firms (fabless semiconductor firms), professional semiconductor processing firms, and packaging and testing firms based on their primary business activities as disclosed in financial statements. The study employs grouped regression to evaluate the impact of trade facilitation on these groups. The regression results displayed in the Column(2) and Column(3) of Table 6 indicate that semiconductor processing and packaging and testing firms, which experience greater inventory pressure, display regression results consistent with the baseline model. However, for design firms, the regression results in column (1) of the Table 6 show that the trade facilitation coefficient is negative and not significant, indicating that improvements in trade facilitation do not significantly increase inventory pressures for firms with lesser demands for non-finished product.

**Table 6:** Regression table by production stage.

VARIABLES	(1)	(2)	(3)
	IC design	IC package	IC fabrication
	Ln(inv)	Ln(inv)	Ln(inv)
Ln(tfi)	-0.195	1.127***	0.582*
	(0.545)	(0.232)	(0.306)
Control Variables	Yes	Yes	Yes
Individual Fixed Effects	Yes	Yes	Yes
Time Fixed Effects	Yes	Yes	Yes
Observations	207	72	180
Number of groups	23	8	20

Note: Standard errors in parentheses;

*** p<0.01,

** p<0.05,

* p<0.1;

The symbol of “ln()” between the variable means the logarithm of this variable.

Wang [17] observes that smaller fabless enterprises rarely receive adequate manufacturing assurances from larger Integrated Device Manufacturers (IDMs) due to inherent conflicts of interest. The growth in capacity and inventory of semiconductor design companies depends on the expansion of manufacturing capabilities, which can lead to a crowding-out effect. As trade facilitation improves, the majority of manufacturing companies on the island, faced with a surge in demand and limited capacity, tend not to prioritize the needs of local small and medium-sized semiconductor design companies. Instead, they first address orders from large semiconductor design companies located elsewhere, thus delaying or reallocating capacity initially intended for smaller firms to entities in supply chains outside the island. Consequently, the inventory levels of semiconductor design companies on the island do not exhibit a significant positive correlation with improvements in trade facilitation levels.

### 4.3 Mediating effect test

The baseline regression demonstrates that higher levels of trade facilitation contribute to increased corporate inventory. This study further explores the market expansion pathway’s role. Hypothesis 2 focuses on whether enhanced trade facilitation boosts market demand for semiconductor companies via this pathway. In this study, bootstrapping and Sobel tests were used to test the mediation effect of market expansion pathway. Table 7 shows that the Sobel test’s z value is significantly at 1% level and that the Confidence Interval (CI) of the indirect effect of the model regardless of the Percentile CI and the bias CI—does not contain 0. For robustness testing, the total value of the electronic component manufacturing industry is employed as mediator variable. After replace the mediator variable with the total value of the electronic component manufacturing industry, the results still keep significantly at 10%. Therefore, it can be determined that the indirect effect is significant, indicating that market size’s predominant mediating role between trade facilitation and corporate inventory, thus supporting Hypothesis 2. Columns (IV→DV) and (IV→M) of Table 8 display regression results, indicating that trade facilitation positively correlates with both corporate inventory and market size, which satisfies the criteria for mediating effect analysis. Analysis of market size’s mediating role in Column (IV+M→DV) shows that while the trade facilitation coefficient becomes insignificant, the market size coefficient remains significant, suggesting market size’s full mediating role between trade facilitation and corporate inventory, thus supporting Hypothesis 2. These results corroborate ZHANG [51]’s findings that market expansion is the most influential factor in how trade facilitation affects corporate inventory, particularly in markets with high product differentiation.

**Table 7:** Sobel test and bootstrap method to test significance of mediation effects.

IV	M	DV	Sobel Test	Bootstrapping 95% CI			
				Percentile CI		Bias CI	
				Lower	Upper	Lower	Upper
Ln(TFI)	Ln(Market)	Ln(Inventory)	0.739***	0.173	1.553	0.151	1.518
Ln(TFI)	Ln(IC_a)	Ln(Inventory)	0.259***	0.029	0.586	0.054	0.663

Note: IV: independent variable; M: mediator; DV: dependent variable; Ln(TFI): the logarithm of Trade facilitation level; Ln(market): the logarithm of Regional market size; Ln(Inventory): the logarithm of Business inventory of non-finished products;

*p<0.1;

**p<0.05;

***p<0.01;

This study employed bootstrapping technique with 5000 bootstraps.

**Table 8:** Results of regression analysis for mediating effects.

IV	M	DV	IV→DV		IV→M		IV+M→DV			
							IV		M	
			ß	S.E.	ß	S.E.	ß	S.E.	ß	S.E.
Ln(TFI)	Ln(Market)	Ln(Inventory)	0.721	0.325**	1.935	0.168***	-0.018	0.367	0.382	0.09***
Ln(TFI)	Ln(IC_a)	Ln(Inventory)	0.721	0.325**	0.402	0.112***	0.443	0.321	0.690	0.139***

Note: IV: independent variable; M: mediator; DV: dependent variable; Ln(TFI): the logarithm of Trade facilitation level; Ln(market): the logarithm of Regional market size; Ln(Inventory): the logarithm of Business inventory of non-finished products; Ln(IC_a): the logarithm of Gross output of the electronic component manufacturing industry;

*p<0.1;

**p<0.05;

***p<0.01.

### 4.4 Moderating effect test

This study examines the moderating influence of geopolitical factors on the effect of trade facilitation on corporate inventory. The research employs the geopolitical risk (GPR) index for Taiwan, developed by Caldara [63], as a moderating variable to evaluate its impact via a yearly average computation of the GPR index [62, 79], which primarily analyzes worldwide geopolitical risks.

The study centralizes the primary explanatory variable (*TFI*_*dt*_) and the moderating variable (*gpr_twn*_*t*_), forming (*c_tfi*), (*c_gpr_twn*), and (*cross_1*) for regression analysis, following the methodology proposed by Hayes [80] and Hayes & Rockwood [81] for testing moderating effects. The findings, detailed in Table 9, reveal insightful patterns.

**Table 9:** Moderating effect regression table.

VARIABLES	(1)	(2)	(3)	(4)
	Ln(inv)	Ln(inv)	Ln(inv)	Ln(inv)
c_Ln(tfi)	1.437***	1.319***	0.492***	0.408**
	(0.275)	(0.298)	(0.117)	(0.137)
c_gpr_twn	2.336***	2.353***	1.169***	1.207***
	(0.112)	(0.106)	(0.190)	(0.178)
cross_1		1.284***		1.093***
		(0.233)		(0.192)
Control Variables	No	No	Yes	Yes
Individual Fixed Effects	Yes	Yes	Yes	Yes
Observations	468	468	468	468
Number of groups	52	52	52	52

Note: Standard errors in parentheses.

*** p<0.01,

** p<0.05,

* p<0.1;

Ln(inv): Logarithmically transformed inventory variable; Ln(tfi): Logarithmically transformed inventory variable; c_Ln(tfi): Centralized Ln(inv) variable; c_gpr_twn: Centralized gpr_twn variable; cross_1: Interaction term between c_ln(tfi) and c_gpr_twn.

**Fig 1:**
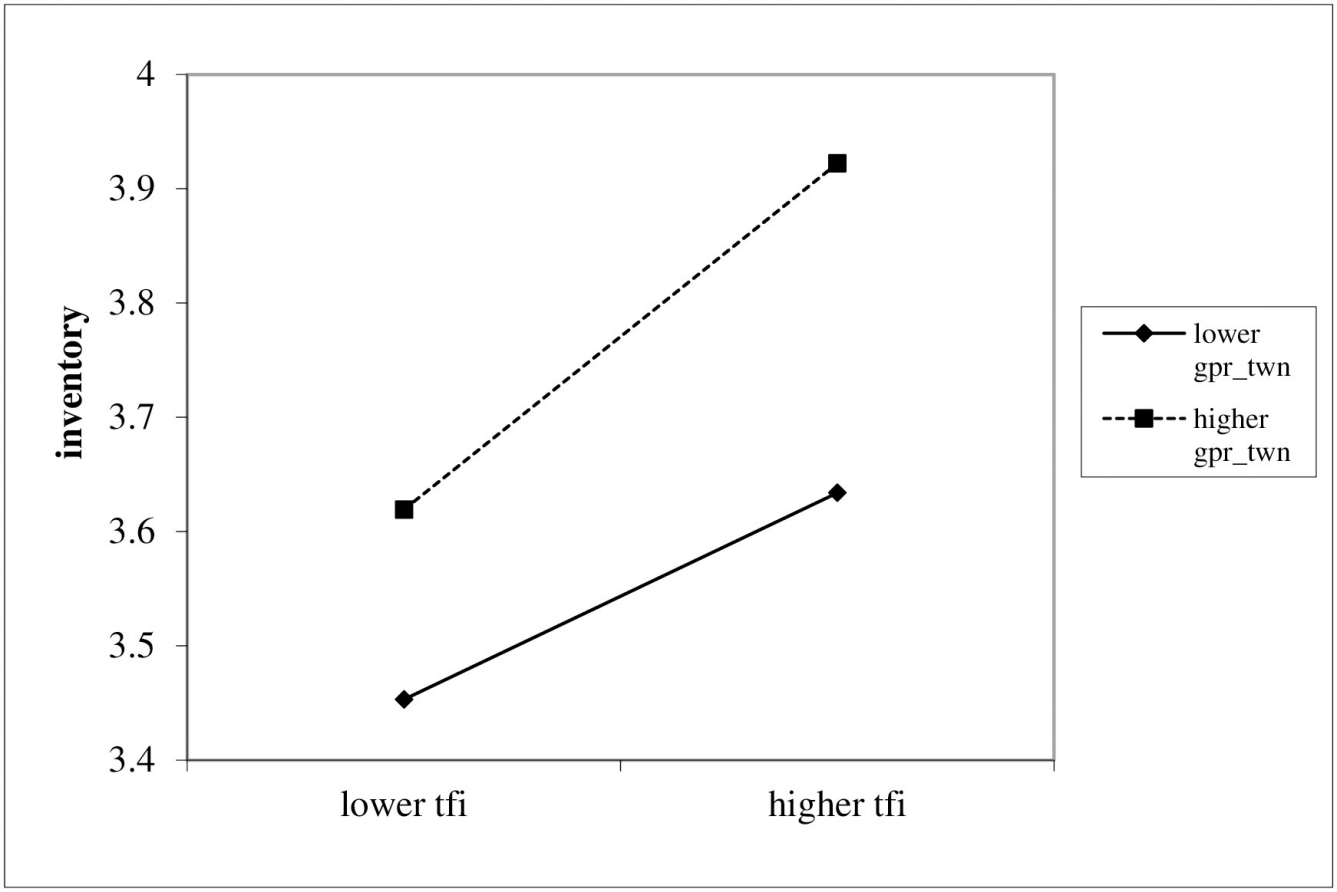
Schematic diagram of regulation effect. Note: the labels for “higher” or “lower” mean these are above or below their corresponding means by one standard deviation, respectively.

First, in Table 9,Column (1) displays the regression results using Driscoll Kraay standard errors with the core explanatory variable, while Column (2) includes the interaction term’s results. The empirical evidence shows a positive coefficient for the interaction term in Column (2), meeting the 1% significance threshold, indicating that geopolitical risks in Taiwan significantly influence the impact of trade facilitation on corporate inventory. Even after introducing control variables in Columns (3) and (4), the main explanatory variable and the interaction term maintain their significance, confirming that geopolitical risks in Taiwan amplify the positive impacts of trade facilitation on inventory management. The results of column (4) show that when the geopolitical risk for Taiwan at the mean value, every 1 unit increase will lead to an average increase of 109.3% in corporate inventory. Hence, Hypothesis 3 was supported. The slope for the link between trade facilitation and corporate inventory moderated by geopolitical risk for Taiwan showed that the relationship became stronger when there was high geopolitical risk for Taiwan. More specifically, as illustrated in Fig 1, when geopolitical risk for Taiwan is high, the impact of trade facilitation on corporate inventory tends to be stronger.

A possible explanation for these findings is twofold. First, from the perspective of strategic inventory, recent deterioration in US-China relations and escalating geopolitical risks in Taiwan have compelled companies to maintain higher levels of strategic inventory to ensure supply chain stability, aligning with Zhang’s [82] observations. Consequently, amid heightened geopolitical risks, companies, under the aegis of enhanced trade facilitation, are able to incur greater costs to sustain strategic inventory as a buffer against uncertainties. Second, from a market size viewpoint, Guanhua [83] points out that due to geopolitical considerations, the West has redirected some security-sensitive orders and capacities to Taiwan, effectively boosting the production, investment, and exports within the island. This study corroborates this perspective and further elucidates that as geopolitical risks intensify, the role of trade facilitation in facilitating this shift in orders becomes more pronounced, thus enhancing the market expansion benefits of trade facilitation. Therefore, increased geopolitical risks significantly amplify the impact of trade facilitation on boosting inventory levels in Taiwanese semiconductor firms.

## 5. Research conclusions

### 5.1 Theoretical contributions

The semiconductor industry is a core area of global technological competition, and the Taiwan region, as an important link in the global semiconductor supply chain, plays a crucial role in maintaining supply chain stability and quickly responding to supply and demand changes amid global economic shifts and geopolitical instabilities. This study uses data from publicly listed integrated circuit companies in Taiwan for empirical analysis and robustly verifies the relationships between trade facilitation, market size, and geopolitical factors using mediating and moderating effect models. The conclusions of this study are two-fold:

Firstly, traditional inventory control models like those proposed by Duan and Jing [28] and Huang [68] suggest that trade facilitation’s impact on corporate inventory primarily operates by reducing both the lead time for company purchases and the uncertainty associated with these lead times. However, in their focus on Chinese industrial companies, highlight heterogeneity in corporate inventory dynamics [28, 68].

In contrast, this study, concentrating on the semiconductor sector, which are characterized by short product life cycles, high product diversity, and limited short-term capacity, discovered through mediating effect tests that market expansion, driven by trade facilitation, is the principal factor influencing inventory fluctuations in Taiwan semiconductor companies. Considering the diversification in semiconductor products and the reduction in their life cycles, the enhancement in trade facilitation leading to market size increase plays a pivotal role in elevating corporate inventory levels. This observation aligns with findings from Zhang [51] and Wan [69], who found that the inventory-augmenting effects of infrastructure development surpass its reduction effects. These conclusions corroborate Wan [69] heterogeneity tests, which indicate that companies with greater output ratios are more susceptible to market expansion effects. Based on these, this study finds that the enhancement of trade facilitation levels in Taiwan predominantly reduces the output delivery costs for semiconductor industries, thus allowing companies to meet larger market demands. This market expansion effect has been validated through a mediating effect test. However, given the semiconductor industry’s limited short-term capacity, the improvements in trade facilitation levels principally bolster inventory for semiconductor companies with manufacturing capabilities and are less impactful for fabless semiconductor design companies.

Secondly, the moderating effect test revealed that shifts in geopolitical factors significantly influence the role of trade facilitation in managing corporate inventory. This finding echoes the research of scholars such as Bown [54], Ramani [16], Tomoo [56], and Yunogami [53], who noted that strained geopolitical relations, exemplified by China-US tensions, lead to persistent trade frictions and the disruption of multilateral international trade norms, thereby severely affecting the degree of trade facilitation. This study finds that under elevated geopolitical risks, the promotion of inventory by trade facilitation in Taiwan’s semiconductor companies is more pronounced. On one hand, under heightened geopolitical risks, the cost reductions achieved through trade facilitation permit companies to maintain higher strategic inventories to ensure supply chain stability. On the other hand, under such risks, some orders and capacities from mainland China are relocated back to production in Taiwan, and the improvements in trade facilitation more effectively enable companies to secure these orders and capacities from the West, thereby further intensifying the inventory pressures on semiconductor companies in the region.

### 5.2 Practical contributions

The findings of this study furnish policymakers with a more comprehensive understanding of trade facilitation measures, providing fresh insights for mitigating inventory pressures and enhancing the supply chain efficiency of semiconductor companies.

Firstly, the research indicates that market size is a significant pathway through which trade facilitation fosters corporate inventory growth. It also shows that semiconductor companies with production capabilities benefit more from the market expansion effects of trade facilitation. When market demand is clearly defined, the positive impact of trade facilitation on corporate supply chain efficiency becomes substantial. Data reveals that Chinese mainland remains Taiwan’s largest export market for electronic components, with Taiwan securing a 35.3% market share of Chinese mainland’s electronic component imports in 2022, similar to the combined market share of Korea, Malaysia, Japan, and Vietnam at 36.4% (https://www.mof.gov.tw/). To more accurately forecast market size in Chinese mainland and align supply capacity accordingly, thereby maximizing the supply chain efficiency benefits derived from trade facilitation, semiconductor companies in Taiwan are encouraged to expand their manufacturing investments in Chinese mainland and strengthen their ties with the mainland’s semiconductor industry innovation network. This strategic approach will leverage the mainland’s market, labor, and land resources, thus diminishing supply chain uncertainties, mitigating the effects of the bullwhip effect, and enhancing the competitiveness and supply efficiency of the cross-strait semiconductor industry.

Secondly, the research underscores that geopolitical risks significantly amplify the market expansion effects of trade facilitation for semiconductor companies. When geopolitical risks are elevated, the inventory pressures on semiconductor companies in Taiwan substantially increase, incurring a range of operational costs that, over the long term, erode the competitive advantage of these companies and lead to a gradual relocalization of capacities involving Taiwan, such as in Japan, which is beginning to enhance international cooperation and reduce its reliance on Taiwan [65] to ensure supply chain security. Therefore, a stable geopolitical environment is essential for the efficient operation and long-term development of local semiconductor companies. Taiwan authorities must adeptly manage cross-strait relations, prevent an escalation in geopolitical risks in Taiwan, and create a conducive environment for businesses.

### 5.3 Research limitations and suggestions

Firstly, the trade facilitation index developed in this study focuses on Taiwan’s counties and cities, not accounting for the impact of trade facilitation levels in other global regions compared to Taiwan. Future studies should broaden the scope and data source of the trade facilitation index to evaluate the performance of various global regions concerning trade facilitation within the semiconductor industry. For example, Financial technology [84] and digital finance [85] data related to e-commerce indicators can be added to further improve the analysis of semiconductor firm supply chain efficiency in the context of digital economy.

Secondly, as semiconductor companies in Taiwan gradually decouple from mainland Chinese enterprises in advanced manufacturing processes and diversify their production capacities, the short-term increase in inventory caused by the relocation of orders back to the island due to rising geopolitical risks may be alleviated. This indicates that the impact of in U.S.-China tensions on trade facilitation and inventory levels may diminish. However, this impact also depends on whether the U.S. intensify sanctions against the semiconductor industry in mainland China and the extent of supply chain cooperation across the Taiwan Strait involving various semiconductor products. Future research should aim to enhance data collection to better understand these industry dynamics.

Thirdly, the limited scope of this paper precludes a thorough examination of the demand factors influencing supply chain efficiency. Subsequent studies might undertake case analyses on elements affecting the efficiency of the integrated circuit industry’s supply chain, specifically determining the impact level of various small and medium enterprises within the upstream and downstream segments of the integrated circuit spectrum. For example, delving into the best practices for deploying AI virtual assistants in internal corporate collaboration environments, and their impact on enhancing production efficiency, risk prediction and demand forecasting of semiconductor firms [86, 87].

Fourthly, this study does not exhaustively detail the varied pathways through which different companies are impacted in examining the moderating role of geopolitical relations on the efficacy of trade facilitation in semiconductor company supply chains. Future research should probe deeper into the resilience and security of semiconductor supply chains under these influences. For example, use a multi-agent system with the switching topologies to model the evolving scenario of a semiconductor supply chain system in which the information flows and/or material flows are changing between the connected case(geopolitical cooperation) and the interrupted case(geopolitical conflict) [88].
