# Correction: Trade facilitation, market size, and supply chain efficiency of Taiwan semiconductor companies

**DOI:** 10.1371/journal.pone.0322506

**Published:** 2025-04-08

**Authors:** The PLOS One Staff

There is an error in the Data Availability statement. The Data source for the China-US relations “http://www.tuiir.tsinghua.edu.cn/info/1145/5980.htm” should have been removed.

The publisher apologizes for the error.
